# Microstructure analysis, tribological correlation properties and strengthening mechanism of graphene reinforced aluminum matrix composites

**DOI:** 10.1038/s41598-022-13793-y

**Published:** 2022-06-10

**Authors:** Fei Wang, Heping Liu, Zesheng Liu, Zhiming Guo, Fenger Sun

**Affiliations:** 1 Southwest Technology and Engineering Research Institute, Chongqing, 400039 People’s Republic of China; 2 School of Material Science and Engineering, North University of China, Taiyuan, 030051 People’s Republic of China; 3 PLA 63850 Troops, Baicheng, 137001 China; 4 School of Mechatronics Engineering, North University of China, Taiyuan, 030051 People’s Republic of China

**Keywords:** Composites, Materials science

## Abstract

In this paper, graphene reinforced aluminum matrix composites are successfully prepared by high-energy ball milling. The results show that no graphene agglomeration is found in the mixed powder. The complex composites prepared by high energy ball milling and powder metallurgy have approximately 4–5 layers of graphene and the thickness of single-layer graphene is approximately 0.334 nm. The final experimental results confirm the formation of compound AlC_3_ in the microstructure, and its diffraction spot index is ($\overline{2 }$00), ($\overline{1 }$1$\overline{1 }$) and (11$\overline{1 }$). The maximum friction coefficient is 0.126, and the average friction coefficient is 0.027, suggesting good wear resistance and corrosion resistance. Additionally, the friction corrosion mechanism of the material is deeply analyzed. The results of strengthening mechanism analysis show that the main strengthening mechanism of the materials designed in this experiment is thermal mismatch strengthening. It can be concluded that the yield strength of the material calculated by the modified model is 227.75 MPa. This value is slightly lower than the calculated value of the general shear lag model (237.68 MPa). However, it is closer to the yield strength value of the actual material (211 MPa).

## Introduction

Aluminum matrix composites are widely used in many fields, such as aerospace, automotive, military and electronic packaging because of their excellent properties^1,2^. The preparation technology of this kind of metal composites has gradually matured, including agitation casting, pressure leaching, friction stirring and powder metallurgy^3^. The strengthening approach of aluminum matrix composites is to add discontinuous hard phase into the matrix in some way. Several popular strengthening phase include ceramic particle, whisker, short fiber and so on^4^. The addition of hard phase to improve the properties of aluminum matrix composites has attracted more and more attention. Therefore, it is of great significance to study aluminum matrix composites improved by hard phase.

With the development of technology, new reinforcement phases are constantly being explored to meet the needs of materials in more fields. Moreover, the desire for some kind of reinforcement can be processed by multidimensional processing technology to enhance the aluminum matrix composite. Since 2004, scientists Geim and Novoselov of the University of Manchester in the United Kingdom have successfully isolated graphene from graphite using micromechanical stripping and described its electronic properties^5^. With the development of science and technology, graphene is favored by an increasing number of researchers, and its application field is also expanding. This is mainly due to graphene's excellent thermoelectric properties, as well as its tensile strength of 130 GPa. Its Young's modulus is 1 TPa, and it has excellent deformation resistance^6,7^. Therefore, graphene has attracted extensive attention in the scientific community. It has been found that graphene is added not only to metallic materials but also to nonmetallic materials. Due to its important influence on the properties of materials, it is widely used in the research and improvement of material properties. Graphene is playing an increasingly important role in the field of materials.

However, graphene has some limitations in its applications. Graphene has a large specific surface area of up to 2630 cm^2^/g^8^, which makes graphene easily agglomerate. If graphene is not uniformly distributed in the matrix, it will have an adverse impact on the properties of the material. To this end, different approaches have been tried to improve the problem and achieve the even dispersion of graphene. Wang et al.^9^ modified aluminum flake powder with mixed graphene oxide nanosheets and polyvinyl alcohol to achieve an effective graphene dispersion in the matrix. The composites are successfully prepared by powder metallurgy and hot extrusion. Xin Gao et al.^10^ achieved uniform adsorption of graphene sheets on aluminum oxide powder through mutual attraction of dissimilar charges. Aluminum matrix composites reinforced with uniformly dispersed graphene were prepared by powder metallurgy. Some urgent problems and deficiencies still restrict its development and application. For example, reduce the weight of the product and ensure the high temperature resistance, bite resistance and wear resistance of the material at the same time.

Herein, it is particularly important to emphasize the fact that there are relatively few studies on the friction corrosion of graphene-reinforced aluminum matrix composites. Most of the research is focused on the friction behavior of aluminum matrix composites. For instance, Manivannan et al.^11^ compared the friction properties of aluminum alloy Al6061 with its nanocomposite through different load frictions. Harun Mindivan et al.^12^ studied the friction properties of different types of aluminum matrix composites reinforced by silicon carbide. As is well known, metal materials are often accompanied by corrosion in a friction environment. For the study of corrosion friction, the composite reinforced material Al-30Fe_3_O_4_-20SiC prepared by Negin Ashraf et al.^13^ improved the corrosion protection efficiency of the matrix to more than 99%. Toptan et al.^14^ studied the corrosion and tribocorrosion behavior of Al–Si–Cu–Mg alloy and its composites reinforced with B_4_C particles in 0.05 M NaCl solution. It is found that the matrix material mainly slides on the B_4_C particles, thus protecting the matrix alloy from serious wear damage. In addition, the strengthening mechanism and microstructure of graphene-reinforced aluminum matrix composites need to be further studied.

In this work, the graphene reinforced aluminum matrix composites (GAMCs) were prepared by high-energy ball milling (HEBM) and powder metallurgy. The hardness and friction corrosion properties of GAMCs were tested. The friction corrosion of graphene aluminum matrix composites is analyzed in detail. Based on previous work, the strengthening mechanism of the material and the main factors affecting the properties of the material are given. The matrix morphology and the distribution of graphene in the grain boundary are observed carefully and the strengthening effect of graphene on the aluminum matrix composite is comprehensively considered.

## Experimental procedure

In this work, Al powder (purity: 99%) was purchased from Tianjin Zhiyuan Chemical Reagent Co., Ltd. The Graphene powder (purity: 96%) came from Qingdao Huagao Graphene Technology Corp. First, uniformly mixed graphene and aluminum powders were prepared by high-energy ball milling at a ball powder ratio of 10:1. The mass of graphene accounts for 0.5%, and the rest is aluminum powder. The milling medium were the stainless-steel ball. HEBM was carried out on a planetary ball mill at a speed of 150 rpm for 2 h. In the second step, 1.5 g of mixed powder was consolidated by cold isostatic pressing. A preform with a diameter of 15 mm and a thickness of 3 mm was formed at a pressure of 750 MPa for 5 min. Finally, the preform is sintered in a vacuum furnace at a heating rate of 4 °C/min to 600 °C, and the holding time is 4 h. After heat preservation, the sample is cooled with the furnace.

The friction and corrosion resistance of the material is tested by electrochemical friction and wear coupling test systems. The open circuit potential (OCP) is the relative value between the working electrode and the reference electrode. It will change with the change in the reference electrode and time. When the electrode interface reaction reaches a steady state, the OCP will also tend to be stable. The test system was a three-electrode system, with high-purity graphite as the auxiliary electrode, composite material as the working electrode and a saturated calomel electrode (SCE) as the reference electrode. The friction length is 5 mm, and the frequency is 0.2 Hz. The initial load is 10 N, and the load is controlled by the weight. The test time was 30 min. The OCP was recorded for 5 min before sliding and 20 min for the intermediate sliding test. At the end of sliding, OCP recording lasted for 5 min. Alumina ball, 6 mm in diameter, can be used as the counterpart. The sample is a cylinder with a diameter of 15 mm and a thickness of 3 mm. Metallographic polishing was carried out before the experiment.

To characterize the morphology of the powders and sintered samples, microstructural analysis was performed using an FEI Quanta FEG 250 scanning electron microscope (SEM) at 20 kV. The phase composition of the matrix and the carbide or oxide is determined by energy dispersive spectroscopy (EDS) and X-ray diffractometry. The microstructure of the GAMCs was observed by high-resolution transmission electron microscopy (HRTEM, JEOL 2000) operating at an accelerating voltage of 300 kV. Thin foil samples were carefully prepared by ion beam 6 milling (Gatan, Model 600, Oxford, UK). An MFT-4000 multifunction material surface performance tester is used to test the friction corrosion of the GAMCs.

To approximately estimate the tensile strength of Al matrix composites, the Vickers hardness was measured by a microhardness tester (HV-1000). The oxide on the surface of the sample was removed with a polishing machine to make the surface smooth. The sample was observed under 400 times magnification through the micrometer eyepiece of the Vickers hardness tester, and then the point to be tested and loaded was selected. The load is 0.98 N, and the loading time is 10 s.

## Results and discussion

### Microstructure analysis

**Figure 1:**
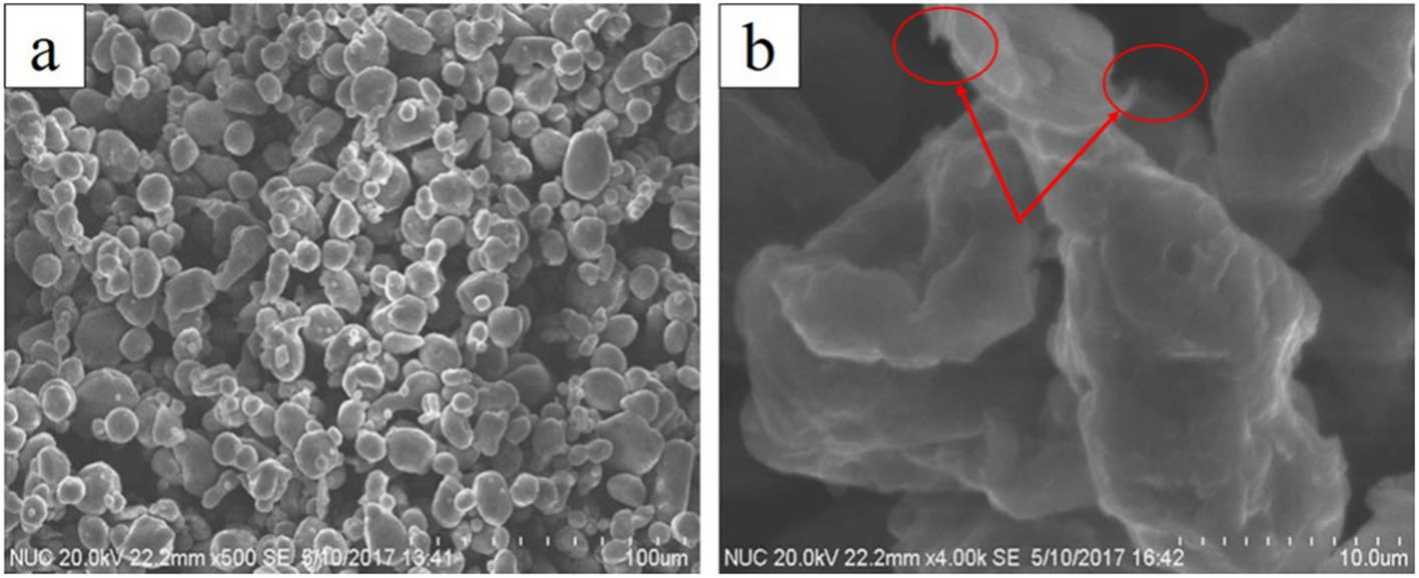
The SEM observation of the mixed powder after grinding (**a**) low multiples; (**b**) high multiples.

SEM observation was conducted on the mixed powder after grinding, as shown in Fig. 1. Most of the aluminum particles in the figure are spherical or elliptical, indicating that there is no obvious coarsening of aluminum powder during ball grinding. The results show that no graphene agglomeration is found in the mixed powder. At high magnification, as shown by the red arrow in Fig. 1b, embedded and suspended graphene were observed on the particle surface. It can be considered that the shear stress of the stainless steel ball causes graphene to embed into the aluminum powder particles during the high-energy ball grinding process, effectively preventing the agglomeration of graphene.

**Figure 2:**
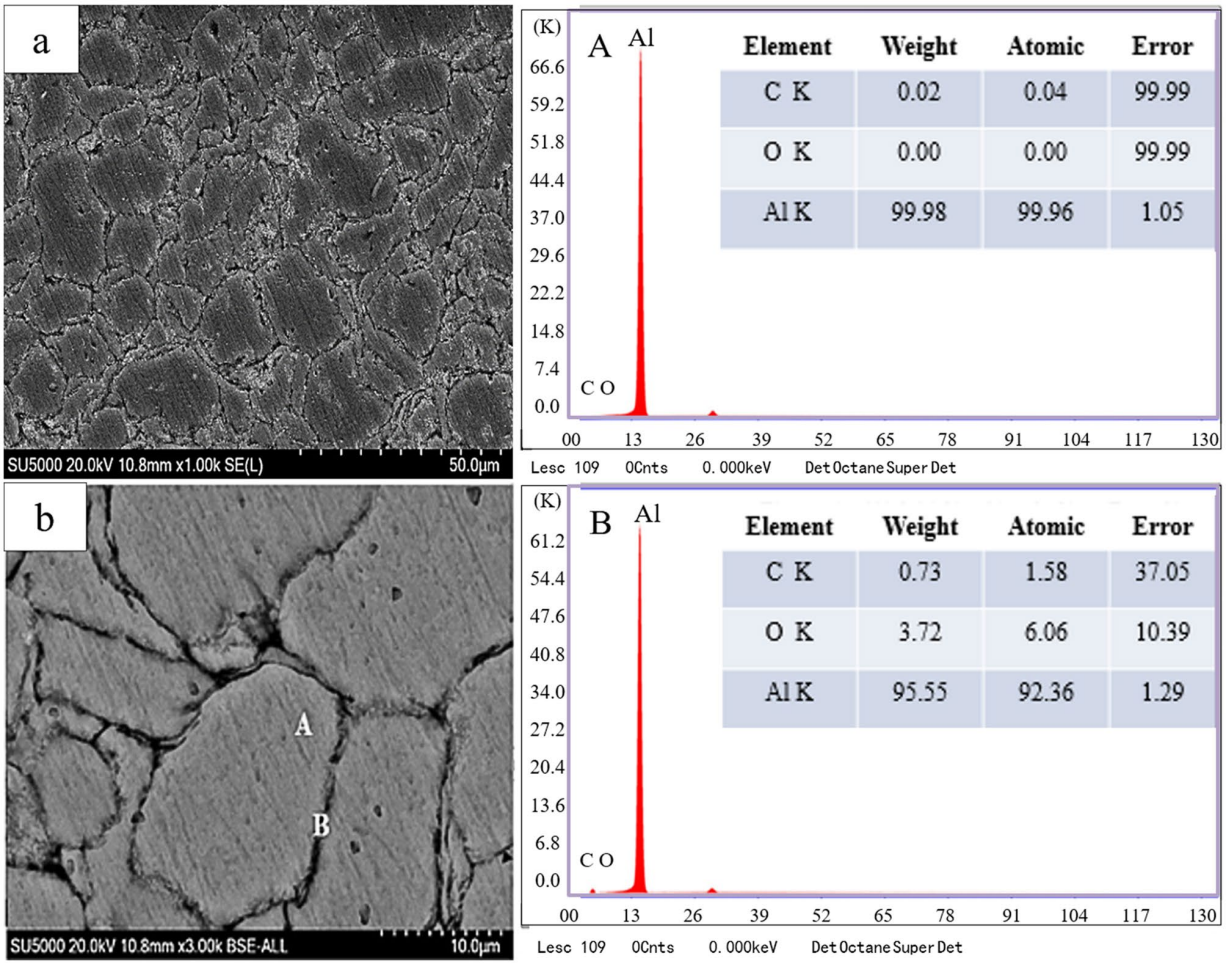
The surface morphology of the prepared GAMCs (**a**) low multiples; (**b**) high multiples.

Moreover, the surface morphology of the prepared GAMCs is observed, as shown in Fig. 2. According to Fig. 2a, the surface morphology at low magnification suggests that the grain size is relatively uniform and that there are no abnormally coarse grains. No cavities or microcracks are found on the surface of the material by high energy ball grinding and powder metallurgy. The surface morphology at high multiples and the interface are tightly bound, as indicated in Fig. 2b. Meanwhile, energy dispersive spectrometry (EDS) is used to analyze the elements and content of the selected location. Point A and point B represent grains and grain boundaries, respectively. Their element distribution and content are clearly visible on the right side of the image. It can be seen that there is only Al and C content at grain A, which proves that there is no oxygen on the grain surface and inside. This kind of situation means that there is no oxidation of aluminum. However, the oxygen content at grain boundary point B is detected as 3.72%, indicating a high probability of alumina formation. Oxygen exists because the air in the powder gap is not easily discharged during the power pressing process.

**Figure 3:**
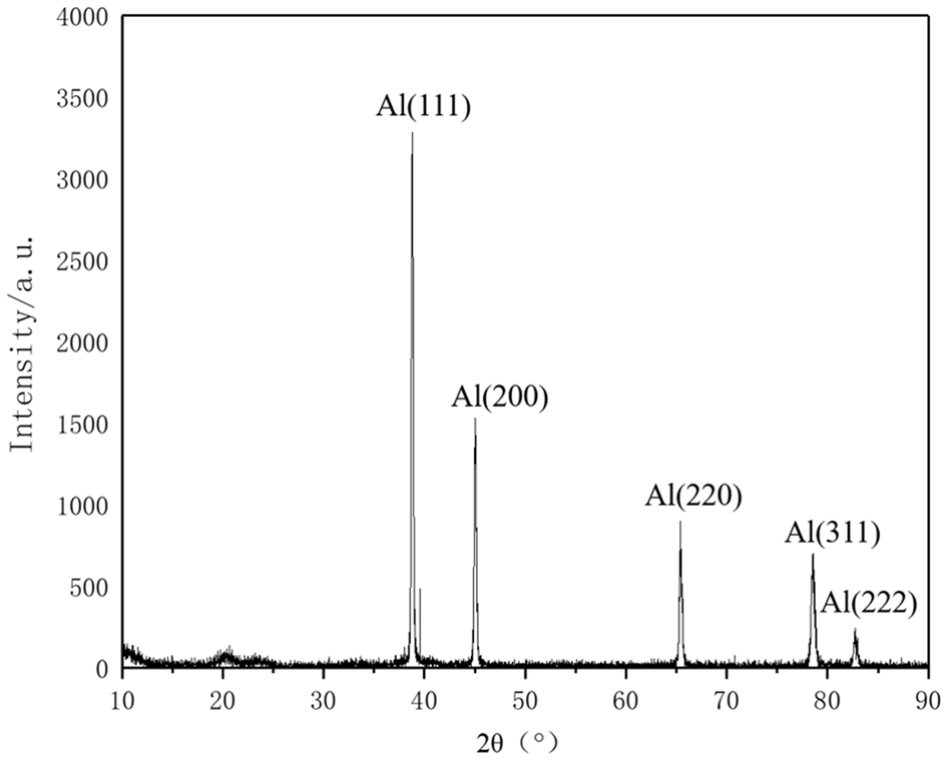
The X-ray diffraction of the sintered sample.

Figure 3 depicts the X-ray diffraction of the sintered sample. The diffraction peaks of the sample are near 38°, 44°, 65°, 78° and 82°, without significant deviation. The corresponding crystal planes are (111), (200), (220), (311) and (222). The absence of a diffraction peak indicates that there is no agglomeration of graphene. The GAMCs are prepared successfully by high energy ball milling and powder metallurgy. At the same time, the peak of alumina is not shown, which is helpful for the next experiment.

**Figure 4:**
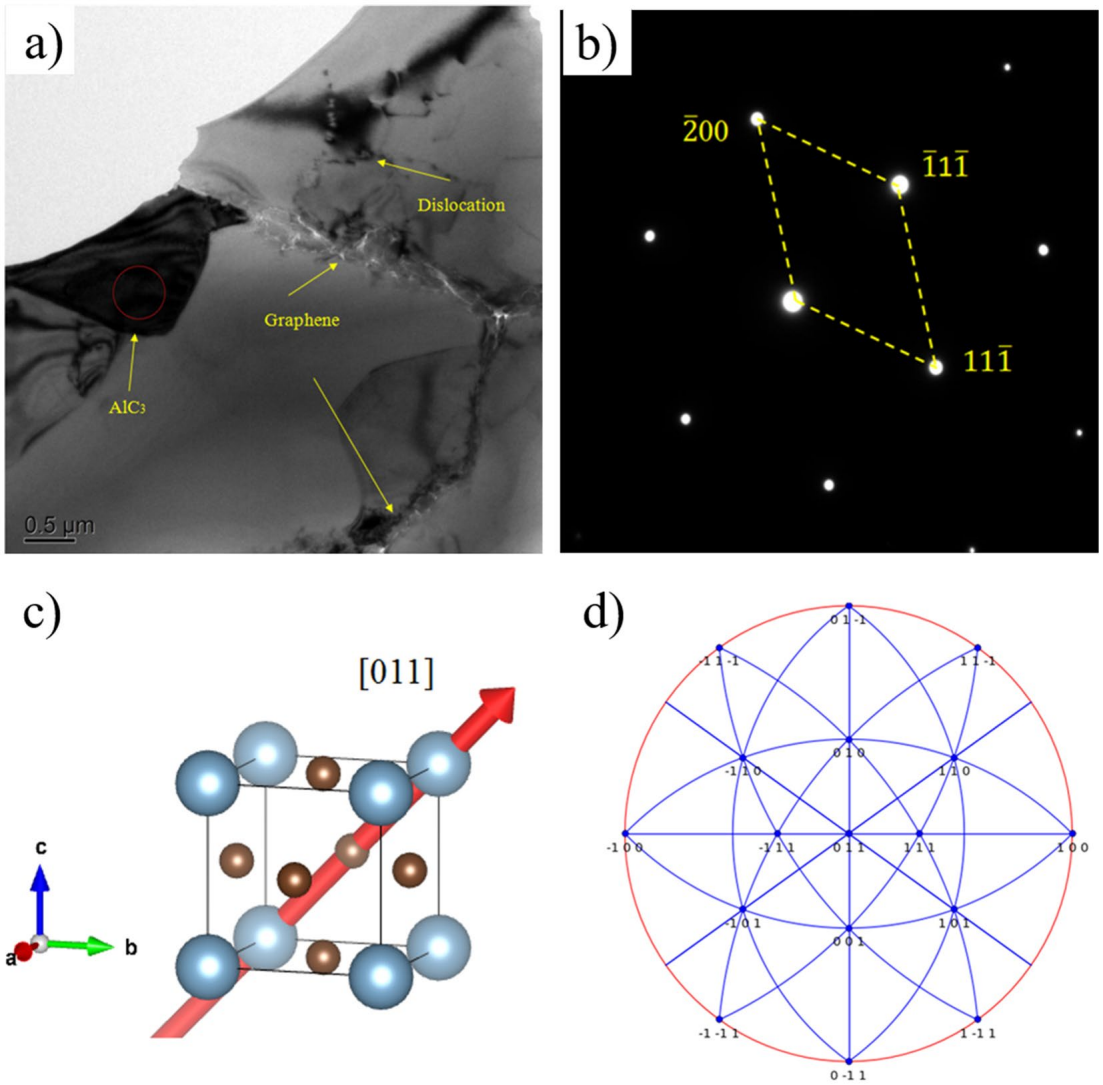
Representative microscopic structure of aluminum matrix composites (**a**) microstructure morphology; (**b**) diffraction pattern of AlC_3_; (**c**) crystal belt axis of [011] direction; (**d**) the stereographic projections of [011] direction.

Figure 4 reveals the representative microscopic structure of aluminum matrix composites. According to Fig. 4a, the gray area is matrix aluminum and the grain boundary is clearly visible. The graphene can be observed at the grain boundary. Some black matter in the matrix may be the product of the reaction between the matrix and graphene or oxygen in the preparation process. To determine its composition, the circular region is subjected to electron diffraction. The selected electron diffraction spots are obtained and the d value is measured. Then, it is compared with the standard card. The results show that the diffraction spots corresponded to the crystal faces of AlC_3_ : ($\overline{2 }$00), ($\overline{1 }$1$\overline{1 }$) and (11$\overline{1 }$), as presented in Fig. 4b. Figure 4c clearly displays the direction of the [110] crystal band axis in the AlC_3_ crystal unit. In addition, it is obvious from Fig. 4d) that the position of the ($\overline{1 }$1$\overline{1 }$) and (11 $\overline{1 }$) crystal planes at the equator plane can be realized more intuitively by using the stereographic projection method. They are located to the left and right of the central axis, respectively. After careful comparison and analysis, the calibrated crystal indices are self-consistent, proving that the black matter is AlC_3_. In the existing research, AlC_3_ is easily produced when most carbon materials are used for the reinforcement of aluminum matrix composites^15–17^. The reaction process of graphene and aluminum can be analyzed from the perspective of thermodynamics. The chemical reaction formula and the relative free energy formula are as follows^18^:

1 $$\frac{4}{{3}}\text{Al+C=}\frac{1}{{3}}{\text{Al}}_{4}{{\text{C}}}_{3}{, \quad \Delta}{\text{G}}_{{\text{Al}}_{4}{{\text{C}}}_{3}}^{\text{free}}{=\Delta}{\text{G}}_{{\text{Al}}_{4}{{\text{C}}}_{3}}^{0}\text{+}\frac{1}{{3}}{\text{RT}} {\text{ ln}}\frac{{\text{a}}_{{\text{Al}}_{4}{{\text{C}}}_{3}}}{{\left({\text{a}}_{\text{Al}}\right)}^{4}}$$

where $\Delta {\mathrm{G}}^{0}$ refers to the standard free energy of formation per mol of carbon, and $\mathrm{a}$ stands for activity, expressed in atomic fractions under ideal conditions^18^. *R* is the ideal gas constant, and *T* is the absolute temperature. Similarly, the chemical reactions and relative free energies of AlC_3_ in the form above can be expressed as follows:

2 $$\frac{1}{{3}}\text{Al+C=}\frac{1}{{3}}{\text{Al}}{\text{C}}_{3}{, \quad \Delta}{\text{G}}_{{\text{Al}}{\text{C}}_{3}}^{\text{free}}{=\Delta}{\text{G}}_{{\text{Al}}{\text{C}}_{3}}^{0}\text{+}\frac{1}{{3}}{\text{RT}} {\text{ ln}}\frac{{\text{a}}_{{\text{Al}}{\text{C}}_{3}}}{{\text{a}}_{\text{Al}}}$$

By comparing Eqs. (1) and (2), it can be observed that the free energy of both is calculated according to the reaction rate of *C* per mole. Therefore, this work does not consider the free energy calculation process of products and only observes the difference in individual values between the two formulas. For every mole of carbon, $\Delta {\mathrm{G}}_{{\mathrm{AlC}}_{3}}^{0}$ is less than $\Delta {\mathrm{G}}_{{\mathrm{Al}}_{4}{\mathrm{C}}_{3}}^{0}$ . *R* and *T* can be considered constant under the same circumstances. Then, the size of the exponential function in the free energy equation can be compared. Since ${\text{a}}_{\text{Al}}$ is determined by the atomic fraction, ${\text{a}}_{\text{Al}}$ should be less than 1. Substituting (1) and (2), then ${\text{ln}}\frac{{\text{a}}_{{\text{Al}}_{4}{{\text{C}}}_{3}}}{{\left({\text{a}}_{\text{Al}}\right)}^{4}}$ should be greater than ${\text{ln}}\frac{{\text{a}}_{{\text{Al}}{\text{C}}_{3}}}{{\text{a}}_{\text{Al}}}$. Therefore, it is known that the free energy of AlC_3_ is less than the free energy of Al_4_C_3_ . Additionally, the formation of carbides is related to the structural integrity of carbon materials^19^. During ball milling, graphene is more likely to form AlC_3_ in the material because of the impact of graphene, which tends to break the graphene bonds. As mentioned above, carbides are not easily formed during ball milling, and if any are formed, the content is very small. This is the reason that the XRD does not detect the carbide peak.

Figure 4 shows that the distribution of graphene powder and grain boundaries does not show obvious agglomeration, indicating that the high-energy ball mill plays a good dispersion role. The graphene sheets at grain boundaries effectively inhibit grain expansion and hinder grain growth. Meanwhile, with its unique structure and excellent mechanical properties, graphene can realize load transfer from the matrix to the interface, effectively improving the mechanical properties of materials^10^. As mentioned in the previous section, graphene has payload transfer during frictional etching and slows down the destruction speed of the matrix surface.

**Figure 5:**
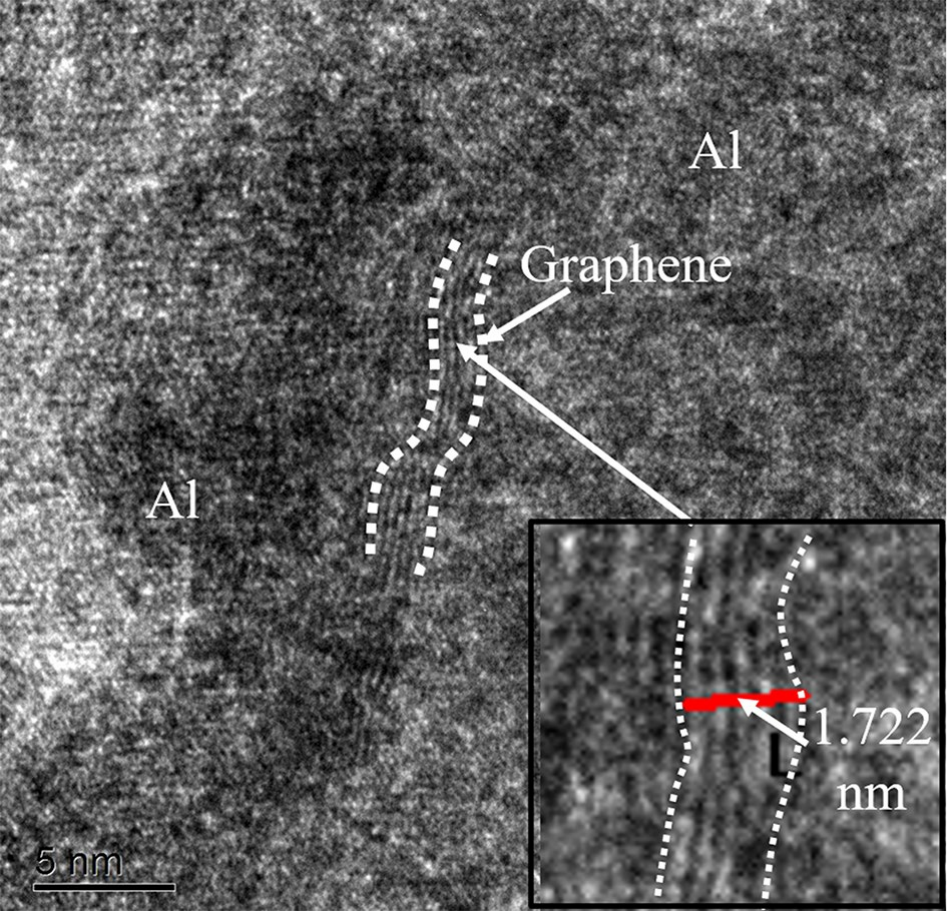
The high-resolution transmission of graphene in this material.

To further study the fine microstructure structure, the high-resolution transmission at the grain boundary is shown in Fig. 5. The distribution of graphene with fewer layers is clearly visible. GAMCs prepared by high energy ball milling and powder metallurgy have approximately 4–5 layers of graphene. By measurement, the thickness of single-layer graphene is approximately 0.334 nm. The shear stress of the stainless-steel grinding ball results in fewer layers of graphene. Graphene aggregation and multilayer graphene are not observed in Fig. 5. The white dotted line shows the distribution of the graphene sheets. The curved distribution of graphene is well adapted to the orientation of grain boundaries. The high matching of graphene orientation and interfacial trend not only promotes the strong interfacial bond between graphene and the matrix but also effectively inhibits the growth of grains.

### Tribological corrosion properties

In the process of preparing and improving aluminum matrix composites, the wear resistance and corrosion resistance of the materials are important indexes to characterize the properties of the materials. Certain metal materials cannot avoid friction and wear during service, such as bearings, cutting tools, drilling equipment and certain precision structures^20–22^. On the one hand, these frictions are conducive to smooth work. They affect the service life of metal materials, production cost and resource utilization^14^. The working environment of most metal materials is exposed to moist air or direct contact with liquid. The flow of air and liquid not only produces friction on the material but also carries out continuous corrosion on it. Due to friction and wear, new contact surfaces are constantly produced on the metal surface, and the contact surface is involved in the corrosion caused by the atmosphere or liquid, which aggravates the wear process. The synergy increases the surface damage rate of metal materials and seriously affects the service life of metal materials^22^.

**Figure 6:**
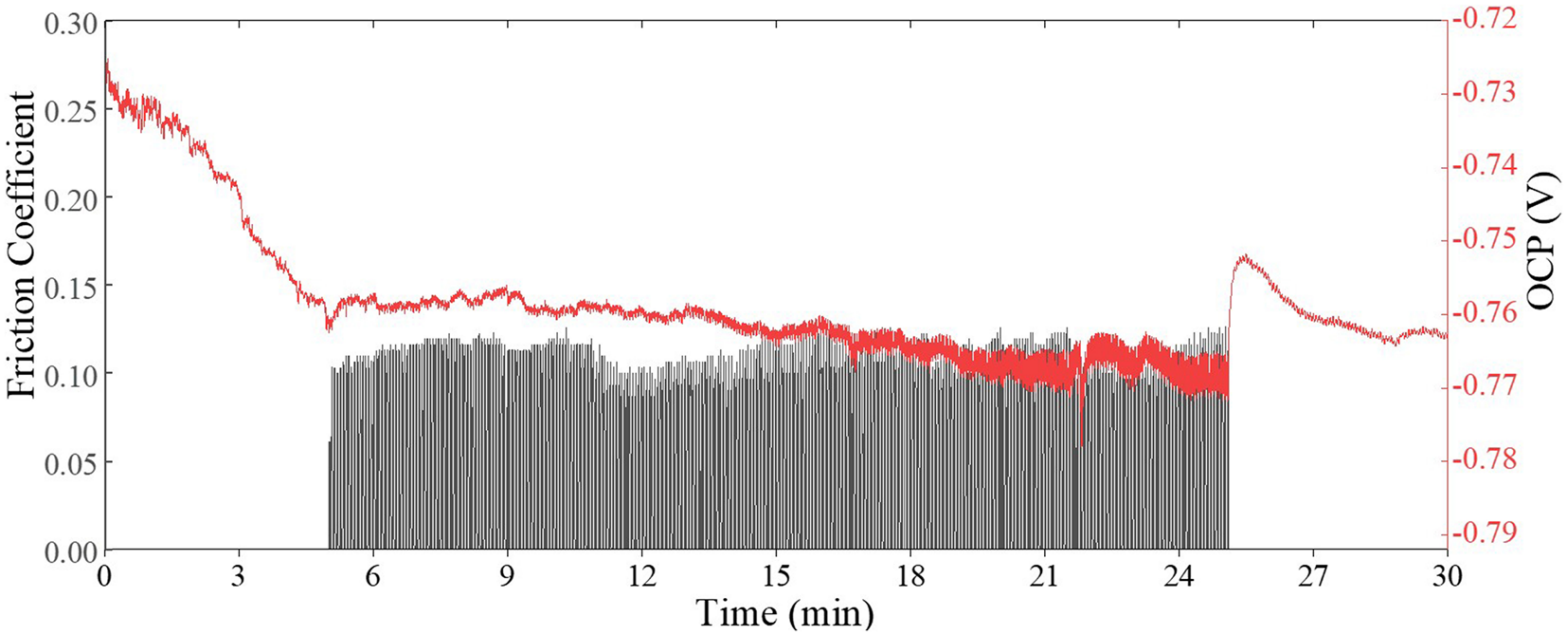
The friction coefficient and OCP of GAMCs over time.

Therefore, to study the friction and corrosion properties of materials, electrochemical friction and wear coupling test systems were used. The friction corrosion process of GAMCs (graphene accounts for 0.5 wt%) is comprehensively analyzed. Figure 6 shows the friction coefficient and OCP of GAMCs during the test. In the initial stage, the friction coefficient increases from approximately 0.10 to approximately 0.11. The maximum friction coefficient is 0.126 and the average friction coefficient is 0.027. On the whole, the friction coefficient of the material is relatively stable.

**Figure 7:**
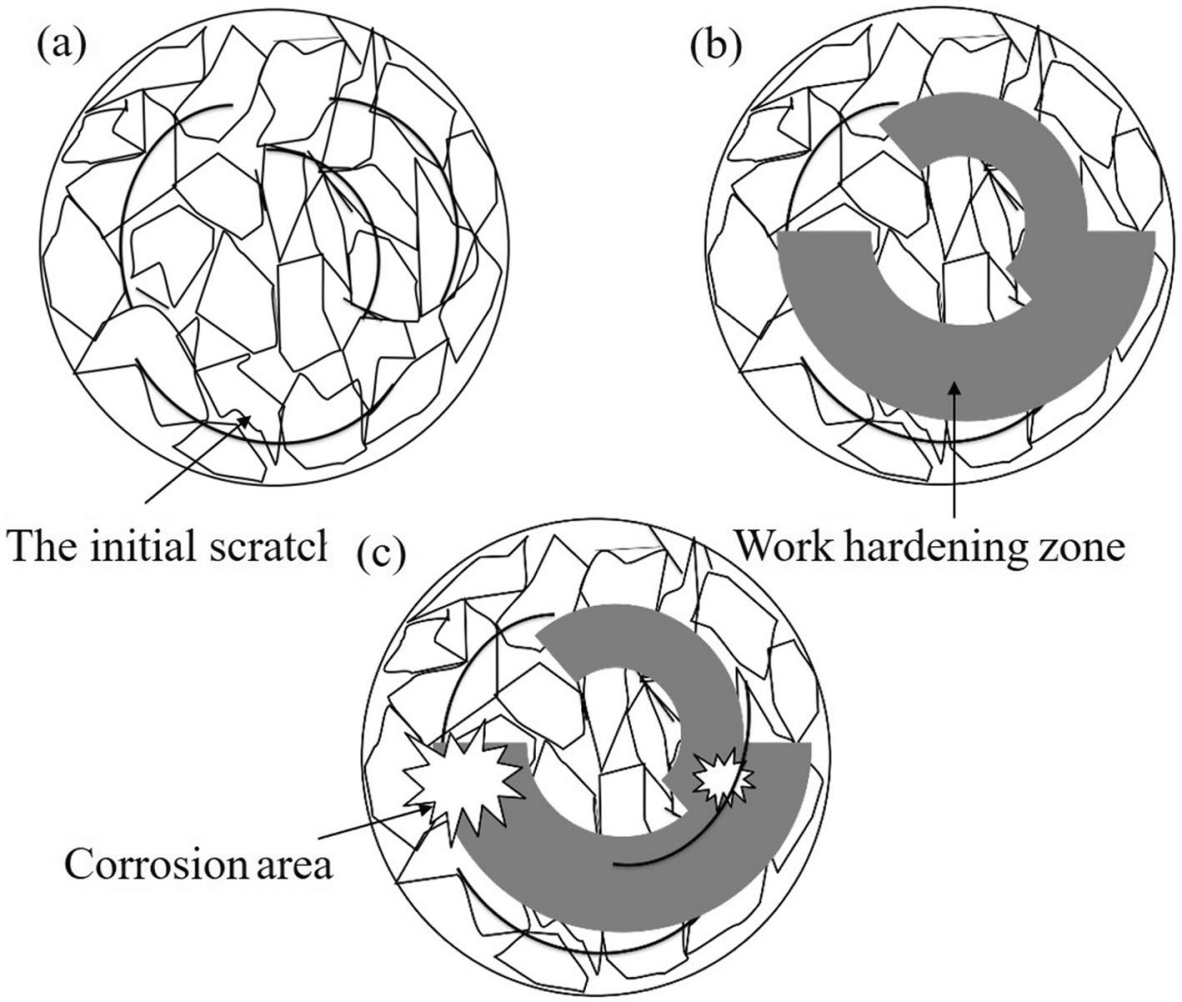
The mechanism diagram of frictional corrosion.

A schematic diagram of frictional corrosion is shown in Fig. 7. In a corrosive environment, aluminum matrix composites tend to react with oxygen in the electrolyte, forming an oxide film that affects the subsequent process^23^. This film can be used as the surface strengthening layer, which is the source point of staggered enrichment and increases the internal stress of the material^24^. The strengthening layer on the initial material surface is not continuous. The friction coefficient tends to rise under the influence of load. As the friction continues, work hardening occurs on the surface of aluminum matrix composites, and the hardening area expands with time^14^. The load action promotes surface strengthening to accumulate more energy, and friction and wear require a greater load. Under the condition of constant load, the friction coefficient of this experiment will be relatively reduced, and the friction coefficient can be as low as 0.10 or below. When the surface energy accumulates to a certain level, corrosion is more likely to occur^25,26^. Tribo-corrosion acts on the material surface to promote surface damage, so that the friction coefficient will be increased again for the next work of hardening. In the case of constant load, frictional corrosion is repeated, and the friction coefficient fluctuates slightly over time, but the average friction coefficient is relatively low.

Frictional corrosion is the joint action of mechanical and chemical friction. Tribological corrosion is defined as degradation due to mechanical wear and corrosion electrochemical etching^14^. Changes in OCP are influenced by this process. A significant decrease in electric potential is observed at the start of the slide as the tribo-etched surface becomes more active under higher normal loads, resulting in lower OCP. In this process, the changes in the surface energy and friction coefficient make the OCP fluctuate slightly throughout the whole process. During the friction phase, the average CPO is approximately − 0.76 V. At the end of tribological corrosion, OCP will have a rising fluctuation due to the formation of a tribological film and the decrease in surface energy.

**Figure 8:**
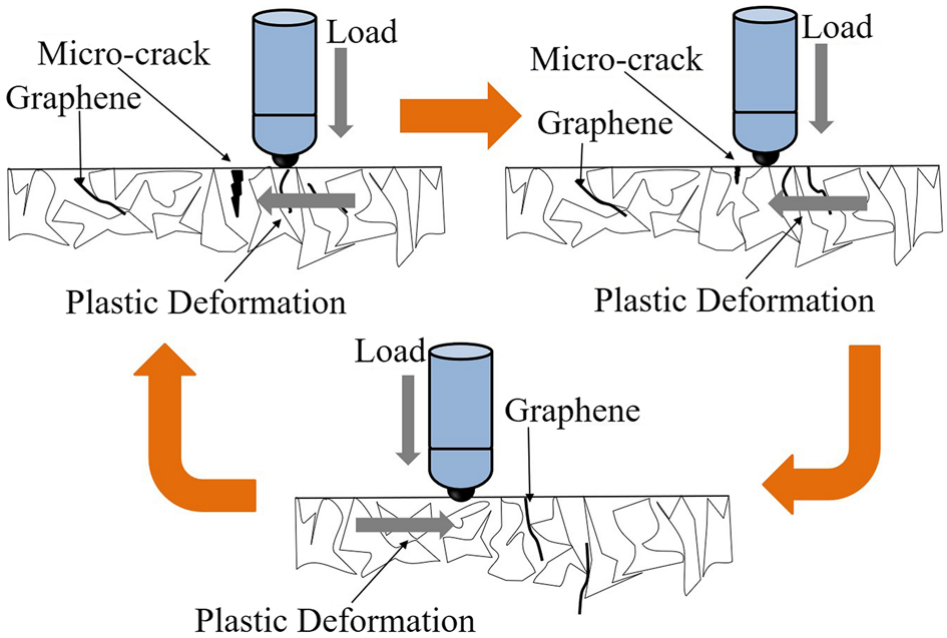
Schematic illustration of the corrosion solution causing micro-cracks in the oxide film area.

Repeated friction of the surface from the alumina ball tends to generate a thin oxide film on the surface, resulting in stress concentration under load. In addition, the action of the corrosion solution causes microcracks in the oxide film area, as shown in Fig. 8. However, the matrix is prone to plastic deformation under high-density loading times^11^. The graphene at and near the grain boundary rapidly transfers the load to the substrate near the surface^27,28^. The plastic deformation near the crack results in the crack being deformed and squeezed, and then the crack is filled and repaired. The repaired crack prevents the electrolyte from entering the matrix, making the frictional corrosion stay only locally, slowing down the corrosion process and reducing the friction coefficient on the material surface. The reciprocating friction of GAMCs is nearly 20,000 times in 30 min. By analyzing the friction data, the friction coefficient and OCP potential of graphene/Al composite materials tend to be stable as a whole. GAMCs do not form in the whole process, which indicates that the material has good friction-corrosion resistance. Graphene is distributed at the grain boundary, which enhances the corrosion and friction resistance of the material because it promotes load transfer and self-repair of the matrix.

### Strengthening mechanism

In this study, the Vickers hardness of pure aluminum was 36 Hv, while that of GAMCs was 61.8 Hv, an increase of 41.7%. The corresponding tensile strength is 211 MPa. During the sintering process, a higher sintering temperature is conducive to nucleation, grain growth, atomic amplitude expansion, atomic diffusion velocity and plastic flow at grain boundaries. The holding time of 4 h makes the atoms at the grain boundary enter the range of atomic force, which strengthens the bonding degree of the grain boundary and obviously improves the strength of the composite.

At present, the strengthening mechanisms of GAMCs are mainly as follows: fine grain strengthening^29^, thermal mismatch strengthening^30^, Orowan strengthening^1^, and shear lag strengthening^31^. This paper will analyze the strengthening effect of GAMCs from the following strengthening mechanisms.

Based on previous studies, the preparation of GAMCs and the binding of graphene at the grain boundary can effectively inhibit grain growth and hinder grain boundary expansion. Hall–Petch^29^ formula is used to calculate the strengthening effect of fine grain strengthening on aluminum matrix composites. The expression is as follows:

3 $${\Delta}{ \sigma}_{\text{G}}\text{=k(}{\text{d}}^{-\frac{1}{{2}}}-{\text{d}}_{0}^{-\frac{1}{{2}}}\text{)}$$

where d is the average grain size of the aluminum matrix composites and ${\mathrm{d}}_{0}$ is the grain size of pure aluminum. Scanning measurement with Image Pro shows that d and ${\mathrm{d}}_{0}$ are 5.62 μm and 11.43 μm, respectively. k is the constant of the influence degree of the grain boundary on the strength (0.04 $\mathrm{MPa}/\sqrt{\mathrm{m}}$^32^).

The enhancement of thermal mismatch is due to the difference in the thermal expansion coefficient between the reinforcement and the matrix when the temperature changes. The effect of this thermal mismatch is more obvious between the normal direction perpendicular to graphene and the matrix, due to the unique two-dimensional structure of graphene. This results in residual thermal stress at the interface between the reinforcement and the matrix, accompanied by the generation of height dislocations. The coefficient of thermal expansion of graphene is 1.1 × 10^–6^ K^−1^, while the coefficient of thermal expansion of aluminum is 23 × 10^–6^ K^−1^, which is an order of magnitude difference. The intensification caused by the expansion difference between graphene and the aluminum base is calculated by using the model formula proposed by Arsenault et al.^33^:

4 $${\Delta \sigma }_{\text{CTE}}= \text{K} {\text{G}}_{\text{m}}{\text{b}}\sqrt{\rho}$$

where ${\text{G}}_{\text{m}}$ represents the shear modulus of aluminum (2.45 × 10^4^ MPa^34^) and K is the constant maturity factor (0.5^35^). b represents the Boggs vector (0.286 nm), and ρ represents the dislocation density. The following formula gives the calculation process of density.

5 $${\rho=}\frac{{12 \Delta {\text{T}} \Delta \alpha }{\text{f}}_{\text{v}}}{\text{(1-}{\text{f}}_{\text{v}}\text{)b}{\text{d}}_{\text{p}}}$$

where ∆T is the temperature change (575 K) and $\Delta \alpha$ is the difference in the coefficient of thermal expansion between graphene and aluminum. ${\text{d}}_{\text{p}}$ is the average surface size of graphene, and ${\text{f}}_{\text{v}}$ is the volume fraction of graphene, which is expressed as follows:

6 $${\mathrm{f}}_{\mathrm{V}}=\frac{{\mathrm{f}}_{\mathrm{m}}/{\uprho }_{\mathrm{GNS}}}{{\mathrm{f}}_{\mathrm{m}}/{\uprho }_{\mathrm{GNS}}+\left(1-{\mathrm{f}}_{\mathrm{m}}\right)/{\uprho }_{\mathrm{Al}}}\times 100\mathrm{\%}$$

Accordingly, Orowan strengthening is also known as second phase strengthening. The graphene is distributed in the matrix as a fine second phase, which can act as a hindrance to dislocation movement. When the matrix is subjected to applied stress, graphene hinders dislocation movement, and the dislocation is bent to form a dislocation ring, thus playing a strengthening role. In this paper, the reinforcement value is calculated using the following model^36^:

7 $${\Delta}{\sigma}_{\text{Oro}}\text{=}\frac{\text{0.13}{\text{G}}_{\text{m}}{\text{b}}}{\lambda}{\text{ln}}\frac{{\text{d}}_{\text{p}}}{\text{2b}}$$

where $\lambda$ is the grain spacing (nm), which can be expressed in the following formula^37^:

8 $${\lambda =}{\text{d}}_{\text{p}}\sqrt{\frac{2}{{3}}}\left(\text{1.25}\sqrt{\frac{\pi}{{\text{f}}_{\text{v}}}}\text{-2}\right)$$

The shear lag model proposed that the strengthening mechanism of graphene-enhanced aluminum matrix composites is that the external load is transferred and dispersed through interfacial shear force. It is believed that the amount of graphene addition and the aspect/diameter ratio of graphene added to the composite material affect the properties of the composites^31^. The yield strength value of GAMCs is calculated by using this model, which is expressed as follows:

9 $${\sigma}_{\text{C}}\text{=}{\sigma}_{0}\text{(}{1}\text{+}{\text{s}}{\text{f}}_{\text{v}}\text{)}$$

where, ${\sigma}_{0}$ is the yield strength of the matrix (150 MPa^1^) and s is the aspect ratio of graphene.

The calculation results of each enhancement effect are shown in Table 1. Due to the large size of the aluminum powder before ball grinding and the short ball grinding time, the aluminum powder is not fully crushed. The grain size is mostly at the micron level, and only a small amount of fine grains exists near the grain boundary. This condition makes the calculated value of fine grain strengthening small, only 9.327 MPa. The distribution of graphene in the matrix is idealized in the calculation of thermal mismatch reinforcement. In addition, the difference in the coefficient of thermal expansion between them is obvious. The calculated result is 42.25 MPa, which tends to be an ideal value. This case shows that the strong effect of graphene under ideal conditions is very considerable, and the preparation process needs to be further improved. In general, graphene has great development value as a reinforcement. In terms of process innovation and preparation, effective dispersion and structural integrity of graphene are the top priorities.

**Table 1:** The calculated results of Graphene/Al composites.

Strengthening mechanisms (MPa)	$\Delta {\sigma }_{G}$	${\Delta {\sigma }}_{\text{CTE}}$	$\Delta {\sigma }_{\mathrm{Oro}}$	${\Delta {\sigma }}_{\text{S}}$
Graphene/Al composites	9.327	45.25	26.9	16.89
Al				150

To further illustrate the reliability of the reinforcement mechanism, the shear-lag model modified by Nardone and Prewo^38^ is used to calculate the yield strength. ${{\varphi }}_{\mathrm{c}}$ is used to denote the yield strength value of GAMCs calculated by the modified model. Then, the expression of the modified model can be expressed as follows:

10 $${\varphi }_{\text{c}}\text{=}\left({{\sigma }}_{0}{+\Delta}{{\sigma }}_{\text{G}}{+}{\Delta{\sigma }}_{\text{CTE}}{+\Delta}{{\sigma }}_{\text{Oro}}\right)\left(\frac{{\text{f}}_{\text{V}}\text{(s+4)}}{4}\text{+1-}{\text{f}}_{\text{V}}\right)$$

Referring to the relevant values, the yield strength of the material calculated by the modified model is 227.75 MPa. Compared with Formula (9), this value is slightly lower than the calculated value of the general shear lag model (237.68 MPa). However, it is closer to the yield strength value of GAMCs (211 MPa), and the experimental results are in good agreement with the calculated results.

## Conclusions

In the current study, graphene reinforced aluminum matrix composites were successfully prepared by a powder metallurgy method. In particularly, the typical microstructure, tribological corrosion properties and strengthening mechanism of this kind of composite are analyzed in depth. The interesting and meaningful innovative results are as follows:

1. According to the experimental results, high-energy ball milling is an effective method to prevent agglomeration of graphene. The complex composites prepared by high-energy ball milling and powder metallurgy have approximately 4–5 layers of graphene and the thickness of single-layer graphene is approximately 0.334 nm. The final experimental results confirm the formation of compound AlC_3_ in the microstructure, and its diffraction spot index is ($\overline{2 }$00), ($\overline{1 }$1$\overline{1 }$) and (11$\overline{1 }$).
2. The maximum friction coefficient is 0.126, and the average friction coefficient is 0.027, suggesting good wear resistance and corrosion resistance. Surface payload transfer and plastic deformation improve the corrosion resistance to a certain extent. The obtained results indicate that the distribution of graphene at grain boundaries plays a role in effective load transfer.
3. The hardness of aluminum matrix composites strengthened by 0.5 wt% graphene prepared by high-energy ball grinding and powder metallurgy reached 61.8 Hv, which is improved by 41.7% compared with pure aluminum, and the strengthening effect is obvious.
4. The strengthening mechanisms of GAMCs are analyzed, and the strengthening effects are calculated. In the calculation process, the distribution of graphene in the matrix is idealized. The thermal expansion coefficients of graphene and aluminum are significantly different, so the thermal mismatch strengthening effect of the calculation is more significant. The yield strength of the material calculated by the modified model is very close to the actual value.

## Data Availability

The datasets generated during and/or analyzed during the current study are available from the corresponding author on request.
